# Myeloid sarcoma in brain and optic nerve presented as a relapse of acute myeloid leukemia: A case report

**DOI:** 10.1002/ccr3.8861

**Published:** 2024-05-07

**Authors:** Mais Musleh, Samer Musleh, Manal Sheikhi

**Affiliations:** ^1^ Department of Hematology, Faculty of Medicine AL‐Assad University Hospital Damascus Syria; ^2^ Department of Neurosurgery, Faculty of Medicine AL‐Abasieen Hospital Damascus Syria

**Keywords:** acute myeloid leukemia, brain, relapse, sarcoma

## Abstract

Myeloid sarcoma (MS) is a rare extramedullary infiltration of acute myeloid leukemia (AML). We present a case of 19‐year‐old male with AML‐M2 who relapse with AML sarcoma in brain and optic nerve. MS as AML extramedullary relapse had a poor prognosis.

## 1 INTRODUCTION

Myeloid sarcoma (MS), also known as chloroma or granulocytic sarcoma, is a condition characterized by abnormal proliferation of blast cells, from one or more myeloid cell, and leads to a disruption in the structure of tissues.^1^ Its incidence ranges from 2.5% to 8%. It is commonly associated with subtypes of acute myeloid leukemia (AML) such as M5a, M5b, M4, and M2.^2^ MS can affect various body parts including lymph nodes, skin, soft tissues, testicles, bones, peritoneum, and gastrointestinal tract.^3^, ^4^ It can occur either on its own or as part of an AML relapse. The diagnosis is typically confirmed through immunohistochemistry analysis of a sample taken from the area. Unfortunately the prognosis of MS is generally poor especially when it is associated with an AML relapse.^5^

## 2 CASE HISTORY

A 19‐year‐old male was diagnosed with AML M2 with a normal karyotype in 2018, and underwent standard treatment with induction 7 + 3, re‐induction 5 + 2, and consolidation therapy (cytarabine 3 g/m^2^). And had achieved complete remission (CR) for 3 years. However, in 2022, the patient began experiencing neurological symptoms, including headaches, loss of vision, weakness in both lower limbs, and difficult to walk.

## 3 METHODS

**FIGURE 1:**
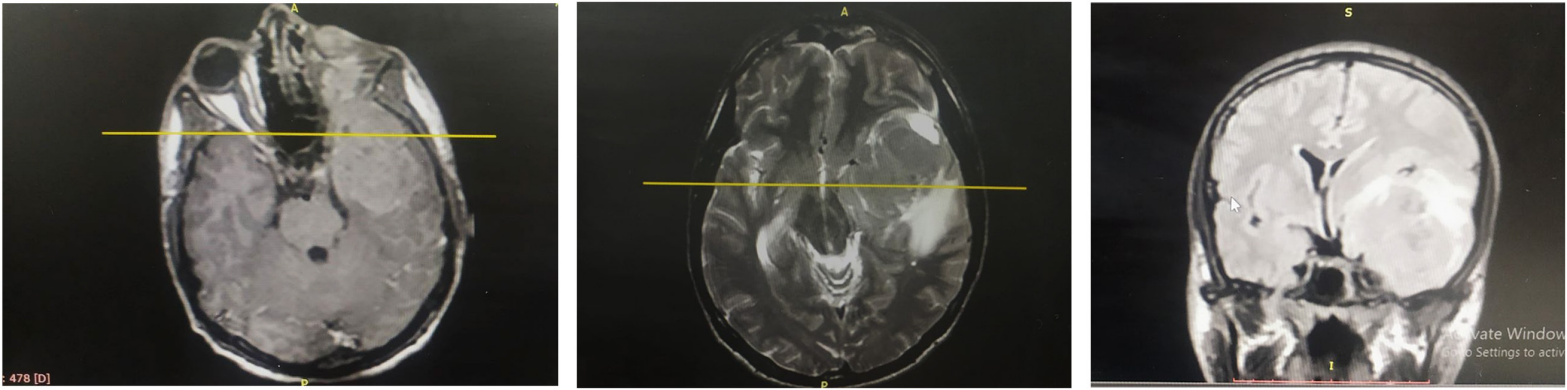
Brain MRI (T1W, T2W, and FLAIR): A heterogeneous mass measuring 9 × 7.5 × 3 cm in the left temporal region, extending to the wing of the sphenoid and causing destruction of the lateral wall of the pilgrims, compressing the middle cerebral artery and infiltrating in the cavernous sinus.

The patient underwent several investigations, including a brain MRI revealed a massive heterogeneous mass 9 × 7.5 × 3 cm in the left sphenoid wing region. This mass exerted pressure on critical structures such as the carotid artery, optic nerve, and cavernous sinus (Figure 1).

**FIGURE 2:**
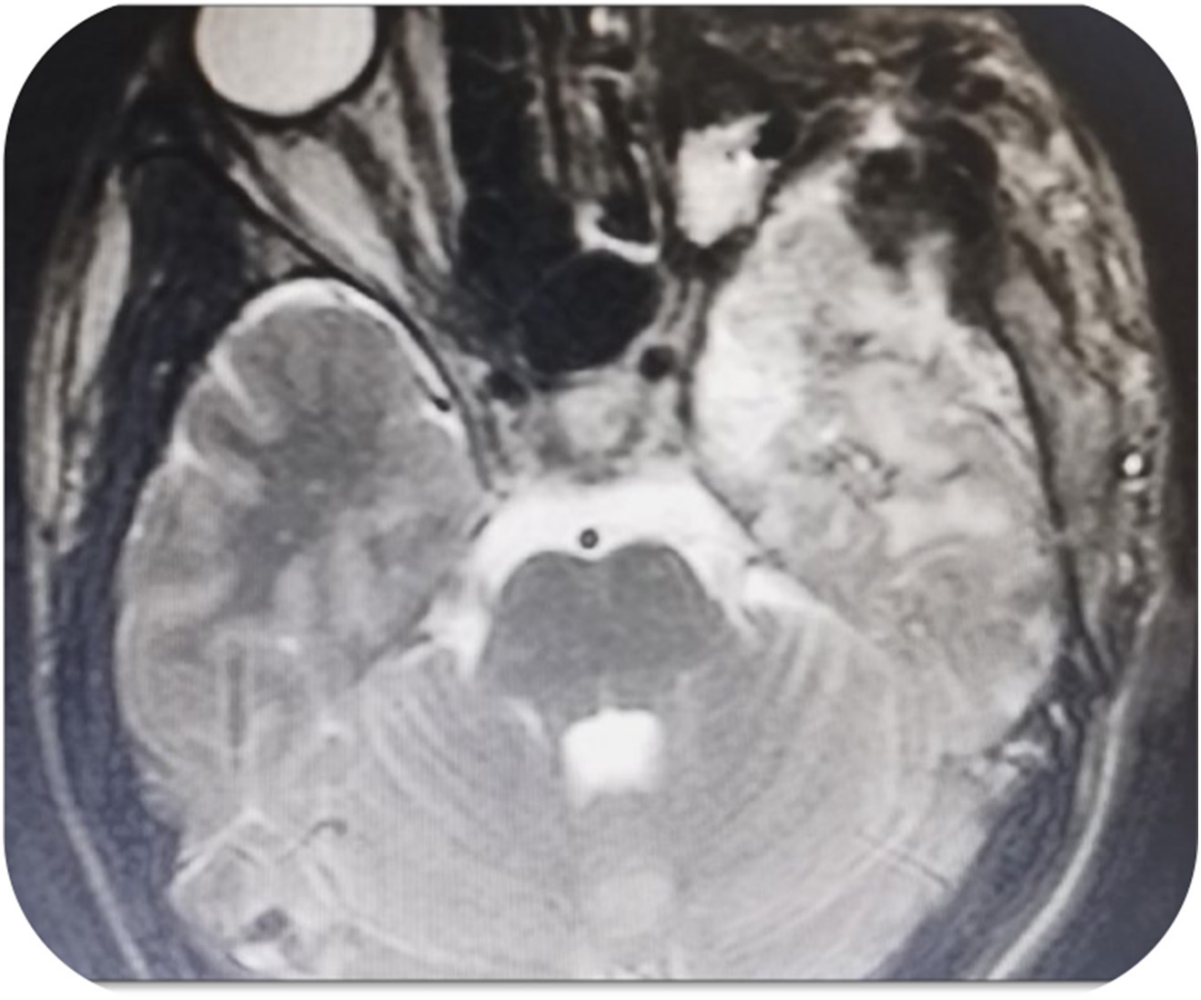
Brain MRI after frontotemporal‐orbit zygomatic (FTOZ) surgery and after resection the mass.

The patient underwent surgery using the frontotemporal‐orbit zygomatic (FTOZ) approach which successfully identified a tumor mass pressing against the carotid artery, median cerebrum, optic nerve, on the left side, optic chiasm, and left cavernous sinus. The patient underwent neurosurgical surgery using the FTOZ approach, which detected a tumor mass compressing the carotid artery, median cerebrum, optic nerve on the left side, optic chiasm, and left cavernous sinus. The tumor mass was removed until intact edges were reached with orbital scraping and optic nerve severing (Figure 2). Pathological analysis of the mass confirmed the presence of a MS involving both the brain and optic nerve, with orbital involvement. Immunohistochemistry further revealed positive staining for LCA, MPO, and CD34, while CD20 and CD3 tests returned negative results. Ki67 proliferative marker showed intermediate activity.

The patient received high‐dose cytarabine (1.5 mg/m^2^) combined with intrathecal injections (cytarabine, dexamethasone, and methotrexate) several times.

## 4 RESULTS AND CONCLUSION

Following treatment, the patient experienced clinical improvement and entered remission. Despite his positive developments, the patient's condition deteriorated, ultimately resulting in his death due to chemotherapy‐related side effects and neutropenic fever 4 months after initiating treatment.

## 5 DISCUSSION

MS is classified as a subtype of AML and related neoplasms by the World Health Organization.^6^ MS is similarly classified by the European Society for Hematology into four separate categories: [1] MS in conjunction with AML, [2] extramedullary relapse of AML, including cases after bone marrow transplantation, [3] blast phase/transformation from myeloproliferative neoplasms or chronic myelomonocytic leukemia, and [4] isolated MS, occurring without a history of myeloid dysplasia and with normal bone marrow aspirate findings.

The incidence of central nervous system (CNS) involvement in MS is relatively rare,^7^ accounting for only 0.4% of cases involving cranial bone marrow, vertebrae, or orbital bones. Its migration to the brain parenchyma is attributed to the disruption of the blood–brain barrier.^8^, ^9^ While orbital involvement as an initial manifestation of AML is uncommon, it is less than 3% of cases.^10^ In our case, brain MRI revealed a heterogeneous mass measuring 9 × 7.5 × 3 cm in the left temporal region, extending into the left sphenoid wing and causing destruction of the pilgrims. The mass compressed the left middle cerebral artery and bulged into the left cavernous sinus (Figure 1). MS can sometimes express B‐cell antigens (CD19 and CD79a), which may lead to misdiagnosis as CNS lymphoma. However, in our case, immunostaining confirmed AML with myeloperoxidase (MPO) positivity.

Treatment approaches for MS lack consensus due to its rarity and limited randomized controlled trials (RCTs). Therapeutic decisions are influenced by factors such as tumor location, the timeline of MS occurrence (before AML onset or AML relapse), patient age, and performance status. Chemotherapy, surgery, radiotherapy, allogeneic hematopoietic stem cell transplantation (allo‐SCT), targeted therapy, and immunotherapy are available therapeutic options.^10^ Surgery plays a vital role in relieving mass effect symptoms, confirming diagnosis, and debulking large‐sized MS before initiating systemic therapy.^3^ In cases of isolated MS with inadequate response to chemotherapy or when rapid relief of vital function impairment is necessary, radiotherapy may be recommended.^1^, ^10^ In our case, we opted for neurosurgical surgery utilizing the FTOZ approach, followed by an induction chemotherapy protocol involving high‐dose cytarabine (1.5 g/m^2^). Cytarabine has a good outcome in MS.

The prognosis of MS remains uncertain due to limited available data. However, it is generally acknowledged that MS occurring concomitantly with AML or as a relapsed AML is associated with a poor prognosis.^11^ Patients who received chemotherapy showed better prognosis compared to those who did not.^12^ The life expectancy of individuals with MS varies based on several factors like age, performance status, and location of the disease, with a reported 5‐year survival rate of approximately 24%.^12^, ^13^ Disease relapse and infections are the most common causes of mortality in MS patients, in our case the patient died with infection after 6 months from diagnosis MS.

In our case, the patient developed MS in multiple organs 3 years after achieving CR from AML, and unfortunately, his condition rapidly deteriorated within 6 months of chemotherapy protocols. This highlights the challenges associated with MS and emphasizes the need for further research and advancements in treatment strategies to improve patient outcomes.

In summary, MS can occur in patients with AML who have been in CR, and can manifest in various organs. Awareness of MS in various organs in relapsed AML is essential, and this diagnosis demands further individualized treatment due to very high mortality risk.

## AUTHOR CONTRIBUTIONS

**Mais Musleh:** Conceptualization; data curation; formal analysis; methodology; project administration; resources; software; writing – original draft; writing – review and editing. **Samer Musleh:** Conceptualization; data curation; formal analysis; investigation; methodology; software; validation; visualization; writing – original draft; writing – review and editing. **Manal Sheikhi:** Conceptualization; data curation; formal analysis; methodology; project administration; resources; software; supervision; writing – original draft; writing – review and editing.

## FUNDING INFORMATION

No funding was obtained for this study.

## CONFLICT OF INTEREST STATEMENT

The authors declare that they have no conflicts of interest.

## ETHICS STATEMENT

No ethical approval was obtained for this study.

## CONSENT

Written informed consent was obtained from the patient for publishing this case report and any accompanying images. A copy of the written consent is available for review by the Editor‐in‐Chief of this journal on request.

## GUARANTOR

Manal Sheikhi is the guarantor of this work.

## Data Availability

All data (of the patient) generated during this study are included in this published article and its supplementary information files.
